# 肿瘤实性成分占比在早期周围型肺癌诊疗中的研究进展

**DOI:** 10.3779/j.issn.1009-3419.2022.102.40

**Published:** 2022-10-20

**Authors:** 汉清 黄, 波 叶

**Affiliations:** 1 525000 茂名，茂名市人民医院胸外科，南方医科大学第一临床学院 Department of Thoracic Surgery, Maoming People's Hospital, The First School of Clinical Medicine, Southern Medical University, Maoming 525000, China; 2 200030 上海，上海市胸科医院胸外科，上海交通大学医学院附属胸科医院 Department of Thoracic Surgery, Shanghai Chest Hospital, Shanghai Jiao Tong University, Shanghai 200030, China

**Keywords:** 肺结节, 肿瘤实性成分占比, 诊断, 治疗, Pulmonary nodule, Consolidation tumor ratio, Diagnosis, Treatment

## Abstract

肿瘤实性成分占比（consolidation tumor ratio, CTR）是近年来肺癌影像学研究的热点，是指在高分辨计算机断层扫描（high resolution computed tomography, HRCT）扫描肺窗中肿瘤最大实性直径与肿瘤最大直径的比值。目前诸多研究也证实其可作为早期肺癌良恶性判断指标及亚肺叶切除的主要参考指标，是早期肺癌复发及预后的独立相关因素。尤其在日本JCOG0804和JCOG0802两个基于肿瘤大小和实性成分占比的研究结果相继公布后，早期肺癌的传统外科手术方式被彻底打破，为早期肺癌患者带来了良好获益。但目前对CTR值的研究数据仍有限，CTR值的测定尚缺少客观的评价系统。本文就CTR值在肺结节良恶性预测、外科手术方式选择、淋巴结清扫、气腔播散（spread through air spaces, STAS）等热点研究方面的进展进行综述，同时进一步探讨CTR值可能存在的疗效预测指标，并对早期周围型肺癌外科治疗的发展趋势以及存在的问题进行总结和分析，为临床工作提供新思路。

## Abstract (en)

Consolidation tumor ratio (CTR) is a hot issue in lung cancer imaging studies in recent years. It is defined as the proportion of the maximum consolidation diameter divided by the maximum tumor diameter in the lung window scanned by high resolution computed tomography (HRCT). Many studies have also confirmed that it can be used as an indicator to identify whether a lung tumor is benign or malignant at the early stage, the main basis on which to decide whether sublobectomy can be performed, and is an independent factor for the recurrence and prognosis of early-stage lung cancer. Especially after tumor size and CTR results of JCOG0804 and JCOG0802 trials in Japan were published, a breakthrough in the treatment method upends the conventional surgical approach, which benefits patients with early-stage lung cancer. But insufficient research data on CTR leads to the fact that an evaluation system to measure CTR is yet to be built. This paper discusses the research progress in CTR prediction of benign or malignancy of pulmonary nodules, how to choose a surgical approach, lymph node dissection, spread through air spaces (STAS) and other hot issues. It also investigates the possible indicators to predict efficacy based on CTR, summarizes and analyzes the development trend of surgical methods to treat early-stage peripheral lung cancer and challenges, to provide new ideas for clinical application.

肺癌是全球最常见的癌症，2020年全球癌症统计中肺癌的发生约占11.4%，其死亡率约占18.0%^[1]^。肺癌患者的5年生存率从ⅠA期的82%下降到Ⅳ期的6%^[2]^。因此，对早期肺癌进行及时的诊断至关重要，其临床治愈率也最高。对早期肺癌的识别，最重要的是评估临床和影像学的危险因素^[3]^。在影像学上对早期周围型肺癌的识别，目前最重要也最热点的是肿瘤实性成分占比（consolidation tumor ratio, CTR）值。它在早期肺癌良恶性的鉴别、外科手术方式的选择等方面发挥着重要作用。本文就CTR值的来源、与影像、病理、淋巴结转移、气腔播散（spread through air spaces, STAS）以及外科手术选择等方面的研究进展进行综述。

## 1 CTR值的来源及在影像学中的研究

在影像学上对肺结节的良恶性的识别和判断常常影响着疾病的治疗和预后。高分辨计算机断层扫描（high resolution computed tomography, HRCT）上肿瘤大小和CTR值对早期周围型肺癌非侵袭性的识别有良好的预测作用。

### 1.1 CTR值来源

在HRCT肺窗中实性成分是指一个浑浊增加的区域，可以完全模糊下面的血管标识；磨玻璃影（ground-glass opacity, GGO）是一个密度轻微均匀增加的区域，该区域不会掩盖下面的血管标记^[4]^。2003年日本学者Ohde等^[5]^提出了在HRCT上测量所有CT切面中发现的肿瘤最大直径（diameter of tumor, Td）、所有CT切面发现的肿瘤最大实变直径（diameter of consolidation, Cdmax），用于计算各最大维度下肿瘤的实变与肿瘤大小之比Cdmax/Td，探索其对区分非侵袭性周围型腺癌的作用。其后为了研究和使用的方便，CTR值被定义为HRCT扫描肺窗中肿瘤最大实变直径与肿瘤最大直径的比值^[6]^。根据肺结节实性成分的多少，我们大致可以把CTR值分为三种：纯磨玻璃肺结节的CRT值为0，部分实性肺结节的CRT值为0＜CTR＜1；纯实性肺结节的CRT值为1。

### 1.2 通过CTR值可以预测肺结节病理的非侵袭性及其预后

Ohde等^[5]^研究发现Cdmax/Td是病理T1N0M0无血管浸润的外周型腺癌的良好预测指标，且Cdmax/Td＜0.5的肿瘤患者具有良好的生存率（5年生存率95.7%）。其后日本临床肿瘤研究小组开展了一项通过HRCT扫描无创预测临床IA期周围型肺癌的病理结果的前瞻性放射学研究（JCOG0201）^[7]^。该研究组定义了影像学上CTR≤0.25的肿瘤为影像学非浸润性肺癌（表明肿瘤具有广泛的GGO区域），而CTR＞0.25为影像学浸润性肺癌。研究者通过对545例临床T1N0M0周围型肺癌患者进行影像学评估后进行肺叶切除和淋巴结清扫手术治疗，术后病理诊断与术前肺癌的影像学诊断进行比较。结果显示：病理性非侵袭性癌可以通过CTR值预测，肿瘤最大直径≤2 cm，CTR≤0.25，对肺癌的特异性为98.7%。该研究平均随访时间是7.1年，随访结果^[8]^认为：在HRCT扫描上肿瘤最大直径≤2 cm、CTR≤0.25和肿瘤最大直径≤3 cm、CTR≤0.5都能很好地无创性预测病理的非侵袭性，这两组患者5年总体生存（overall survival, OS）率均约为97%。

## 2 CTR值在早期周围型肺癌外科手术中的研究

1995年北美肺癌研究小组的一项前瞻性随机试验^[9]^奠定了肺叶切除+肺门纵隔淋巴结清扫为周围型T1N0非小细胞肺癌（non-small cell lung cancer, NSCLC）的首选手术方式。但是基于肿瘤大小和CTR值的亚肺叶切除的临床研究探索^[10]^，极大地改变了这一现状，并为外科手术的选择和治疗带来便利。其中最著名的研究是JCOG0804、JCOG1211和JCOG0802研究（表 1）。

**表 1:** 日本JCOG0804、JCOG1211、JCOG0802临床研究总结 Summary of clinical studies of JCOG0804, JCOG1211 and JCOG0802 in Japan

Project name	Inclusion criteria	Surgical method	Lymph node dissection	5-yr RFS	5-yr OS	Local recurrence	Recommended method
JCOG 0804	T≤2 cm and CTR≤0.25	Segmentectomy/edge resections	Not mandatory	99.7%	99.7%	No	Segmentectomy/ wedge resections
JCOG 1211	(2 cm＜T≤3 cm，and CTR≤0.5)/ (T≤2 cm and 0.25＜CTR≤0.5)	Segmentectomy	Hilar, interlobar and intrapulmonary	98%	98%	-	-
JCOG 0802	T≤2.0 cm and CTR＞0.5	Segmentectomy/Lobectomy	Hilar and mediastinal	Segmentectomy *vs* Lobectomy: 88% *vs* 87.9%	Segmentectomy *vs* Lobectomy: 94.3% *vs* 91.1%	Segmentectomy *vs* Lobectomy: 10.5% *vs* 5.4%	Segmentectomy
T: maximum tumor diameter; CTR: consolidation tumor ratio; RFS: relapse-free survival; OS: overall survival.							

JCOG0804研究^[11]^建议：肿瘤位于肺外周1/3、最大直径≤2 cm且CTR≤0.25，在保证足够切缘（至少5 mm，中位病理手术切缘为15 mm）推荐亚肺叶（肺段/楔形）切除作为首选手术方式。主要终点是5年无复发生存率（relapse-free survival, RFS）。该研究结果显示：290例患者5年RFS为99.7%，且未出现局部复发事件，仅有1例死于其他疾病。中位随访时间为5.5年。从亚组分析来看，楔形切除组5年RFS达100%，肺段切除组有1例死于其他疾病。

JCOG1211研究是一项多机构非随机验证性试验^[12]^。主要入组标准为：肿瘤最大直径＞2 cm但≤3 cm，且CTR≤0.5；或者肿瘤最大直径≤2 cm，CTR＞0.25但＜0.5；肿瘤位于肺外周1/3。主要研究终点是所有行肺段切除术+肺门淋巴结清扫的患者的5年RFS。研究最终结果尚未正式发表，但2021年欧洲心胸外科大会上有研究者展示了该研究中154例肿瘤最大直径＞2 cm但≤3 cm且CTR≤0.5的患者5年OS和RFS均为98%。

JCOG0802研究^[13]^认为对于肿瘤最大直径≤2 cm、CTR＞0.5的周围型NSCLC，应采用肺段切除术而不是肺叶切除术。研究终点为患者OS，是一项非劣效性研究。方法是在标准治疗组进行肺叶切除术、肺门和纵隔淋巴结清扫。在实验治疗组中进行肺段切除术加肺门和纵隔淋巴结清扫。研究结果^[13]^已正式发表：5年OS：肺段切除组（94.3%）＞肺叶切除组（91.1%）；5年RFS：肺段切除组（88.0%）和肺叶切除组（87.9%）无差异；局部复发率：肺段切除组（10.5%）＞肺叶切除组（5.4%）；其他癌症相关死亡：肺叶切除组＞肺段切除组。肺功能方面：第一秒用力呼气量（forced expiratory volume in one second, FEV_1_）（6个月和1年）：肺段切除下降10.4%和8.5%，优于肺叶切除的13.1%和12%。

## 3 CTR值在早期肺癌病理中的研究

CTR值在早期肺癌病理类型、Ki-67的表达、预测STAS等的研究中起作用。

### 3.1 CTR值不同提示肿瘤类型及病变程度不同

随着CTR值的升高，肿瘤可能出现浸润的概率也越来越大。从癌前病变到出现浸润通常是一个连续的过程，从浸润前非典型腺瘤性增生（atypical adenomatous hyperplasia, AAH）和原位腺癌（adenocarcinoma *in situ*, AIS）到微浸润腺癌（minimally invasive adenocarcinoma, MIA），然后是浸润性肺腺癌^[15]^。JCOG0201研究^[4]^显示肿瘤最大直径≤2 cm、CTR≤0.25中98.7%的结节病理为非浸润性癌。病理学特征分析^[6]^上发现GGO组大多数患者是肺腺癌（99.3%），而CTR=1实体结节组中发现了其他组织学类型且经常出现低分化病变。有研究^[16, 17]^表明CTR值越大，浸润成分越多，病理有实性或微乳头状成分的机会越大，预后不佳；CTR＞27.4%会提示磨玻璃结节中为非附壁生长为主的浸润性肺腺癌。

### 3.2 CTR值与Ki-67的高表达显著相关

北京大学中日友好医院Liu等^[18]^通过回顾性分析周围型IA期肺腺癌376例患者临床病理特征认为：CTR值与Ki-67的高表达显著相关。

### 3.3 CTR值可以预测STAS

2021年第五版世界卫生组织肺、胸膜、胸腺和心脏肿瘤分类已明确STAS是具有预后意义的组织学特征。有多项研究^[19-22]^表明：STAS的存在与最大肿瘤直径、最大实性成分直径和CTR值有关，在CTR值较高的肺癌中比在CTR值较低的腺癌中更常见。周围GGO的存在与STAS阳性腺癌呈负相关，GGO的缺失与STAS发生独立相关。韩国学者Kim等^[23]^研究发现：STAS在纯实性病变（CTR=1）中比在部分实性病变（0＜CTR＜1）中大约多3倍，而在纯磨玻璃性病变（CTR=1）中不存在。其后进一步的分析提示：STAS存在于CTR＞0.4的肿瘤中，而CTR＜0.4的肿瘤中未发现STAS。

## 4 CTR值与淋巴结转移间的关系

CTR值对于早期周围型肺癌是否需要进行淋巴结清扫以及清扫的范围有着重要的参考价值。CTR值是淋巴结转移的独立因素，当CTR＜0.5可以考虑避免淋巴结清扫。

### 4.1 CTR值是淋巴结转移的独立因素

目前有多项研究^[24, 25]^表明CTR值是淋巴结转移的重要预测因子，但对于CTR值的界定，受到肿瘤大小、胸膜侵犯以及病理类型的影响。日本学者Koike^[26]^分析淋巴结转移的预测因子中发现，当肿瘤最大直径≥2.0 cm、CTR≥89%时纵隔淋巴结转移的概率为33.8%。北京大学中日友好医院Shao等^[27]^研究认为：CTR值是淋巴结转移的独立因素。如果肿瘤CTR≥79.50%或者脏层胸膜侵犯建议行系统淋巴结清扫术，并且建议14个淋巴结作为评估淋巴结检查的切入点。北京协和医学院肿瘤医院Zhao等^[28]^回顾性研究中分析了2,504例部分实性结节患者，其研究结果认为：微血管侵犯、微乳头状/实性亚型和CTR＞0.61与淋巴结转移独立相关（*P*＜0.01）。

### 4.2 CTR＜0.5可以考虑避免淋巴结清扫

淋巴结清扫是肺癌手术一项重要内容，目前基于影像学检查指导淋巴结的研究一直以回顾性分析为主，众多的研究结论认为CTR＜0.5可以考虑避免淋巴结清扫。国立台湾大学医院Tsai等^[29]^分析352例接受标准肺叶切除和淋巴结清扫的肺腺癌患者发现对于肿瘤最大直径＜1 cm、CTR＜0.5的患者，避免标准的肺叶切除或淋巴结清扫可能是合理的。日本学者Suzuki等^[30]^研究发现，含有GGO成分的（CTR≤0.5）肺癌，即使肿瘤超过3.0 cm，也没有淋巴结受累。上海复旦大学附属肿瘤医院Zhang等^[31]^回顾性分析2,749例接受肺切除术并系统淋巴结清扫的侵袭性NSCLC患者发现CTR≤0.5磨玻璃结节均无淋巴结转移。而当CTR＞0.5时，淋巴结转移的发生率显著升高。对于前瞻性临床研究，目前复旦大学附属肿瘤医院一项基于影像学检查来指导淋巴结清扫范围的前瞻性观察临床试验（NCT03216551）正在进行中。

## 5 CTR值对早期肺腺癌患者预后的关系

HRCT中肿瘤最大直径、CTR值是预测磨玻璃样NSCLC患者肿瘤侵袭性和预后的简单而有用的工具^[32]^。CTR值与早期肺癌患者的OS和RFS密切相关，CTR≤0.5常常提示着良好的预后。目前多项研究^[33-35]^发现CTR值是临床IA期肺腺癌的一个独立预后因素，在含有GGO的肺结节中，GGO的存在和CTR值的大小与生存率显著相关。Fu等^[36]^研究发现在亚厘米（最大直径≤8 mm）纯GGO病变中未发现淋巴结转移，CTR值可用于区分患者OS，单纯GGO病变的5年OS良好。Suzuk等^[37]^研究发现cT1N0M0肺腺癌患者的CTR≤0.5和（或）实性成分≤10 mm的复发率极低，手术切除的治愈性极大。日本学者Ito等^[38]^收集了543例肺叶切除的肺腺癌患者的10年OS和RFS，其研究发现：影像学上肿瘤最大直径为2 cm、CTR≤0.25的患者无一复发。肿瘤最大直径为3 cm、CTR≤0.5也有着良好的预后相关。研究者根据肿瘤CTR值和肿瘤大小将患者分为4组。A组和B组包括CTR≤0.5和肿瘤最大直径≤3 cm；A组包括肿瘤最大直径≤2 cm且CTR≤0.25，B组为剩余肿瘤。C组和D组均为CTR＞0.5的肿瘤。C组为肿瘤最大直径≤2 cm，D组为最大直径2 cm-3 cm的肿瘤。10年OS为80.4%，10年RFS为77.1%。A组的10年OS为94.0%，B组为92.7%，C组为84.1%，D组为68.8%，各组的10年RFS分别为94.0%、89.0%、79.7%和66.1%（表 2）。

**表 2:** 临床T1N0肺癌患者肺叶切除术后的长期生存结果 Long-term survival outcomes after lobectomy in patients with clinical T1N0 lung cancer

Group	Tumor size (cm)	CTR	10-yr OS	10-yr RFS
A	≤2	≤0.25	94%	94.0%
B	2-3	≤0.5	92.7%	89.0%
	＜2	0.25-0.5		
C	≤2	＞0.5	84.1%	79.7%
D	2-3	＞0.5	68.8%	66.1%
Group A: tumors with CTR＜0.5 and tumor size ≤2 cm with CTR＜0.25; Group B: tumors with CTR≤0.5 excluded from Group A; Group C: tumors with CTR＞0.5 and tumor size≤2 cm; Group D: tumors with CTR＞0.5 and tumor size 2 cm to 3 cm.				

## 6 总结和分析

CTR值是目前常用的影像学观察指标，基于影像学的指导对早期周围型肺癌的外科手术选择具有重要的参考价值。在保证手术足够切缘情况采纳JCOG0804研究结果治疗早期周围型肺癌已得到众多胸外科医生的认可。而对于近期发表的JCOG0802研究结论：肿瘤直径≤2 cm、CTR＞0.5的外周型NSCLC，应采用肺段切除术而不是肺叶切除术。这一结果目前在国内仍未达成共识，争议较大。考虑肺段切除对于肺功能保护的远期效果并不明显，国内早期周围型肺癌人群庞大对于肺段切除的高复发率暂时难被大众接受等原因，对于该组患者我们仍认为应该选择肺叶切除+肺门纵隔淋巴结清扫作为标准术式。类似的研究北美CALGB/ALLIANCE 140503研究^[39]^正在进行，我们期待未来可以有更多的证据支持肺段切除术作为替代。

淋巴结清扫和肿瘤术后复发一直是胸外科关注点。对于早期周围型肺癌的淋巴结清扫现阶段仍未形成共识。建议CTR值可作为重要参考。对于CTR＜0.5早期周围型肺癌无需行系统淋巴结清扫，淋巴结采样应该是足够的。而当CTR＞0.5时，淋巴结转移的发生率显著升高，建议行系统淋巴结清扫。对于早期肿瘤复发目前有多项研究^[40, 41]^显示早期肺癌手术切除术后5年以上仍有可能会复发，我们建议对于早期浸润性肺癌患者在手术后5年仍需继续观察随诊。

磨玻璃样结节具有惰性生长，Kakinuma等^[42]^发现纯磨玻璃结节发展为部分实性结节的平均时间为（3.8±2.0）年。对于磨玻璃样肺结节手术干预的最佳时机一直未能达成共识。2020年6月开始日本启动了一项多机构、单臂、开放性、非随机的验证性临床试验JCOG1906（EVERGREEN研究）^[43]^，对肿瘤最大直径≤2 cm、CTR≤0.25周围型肺结节进行最长10年的影像学观察。主要研究终点是第二次登记的所有患者的10年OS。希望以此提醒不要在没有确凿科学证据的情况下进行手术。

CTR值的研究给临床工作带来方便的同时也存在一些争议的地方：①CTR值取决于主观评价，目前缺少客观的评价系统；②CT的实性成分与病理浸润性并不完全对应；③CTR值与当前肿瘤原发灶-淋巴结-远处转移（tumor-node-metastasis, TNM）分期中的T期并不能完全对应；④肿瘤直径≤2 cm、CTR≤0.25的结节是否需要手术以及手术的时机等问题。期待后续能有更多的证据来完善从而使早期周围型肺癌的诊断和治疗更加严谨和规范。
